# Impact of a modified progressive Copenhagen adduction exercise programme on hip adduction strength and postexercise muscle soreness in professional footballers

**DOI:** 10.1136/bmjsem-2019-000570

**Published:** 2019-10-15

**Authors:** George Polglass, Adam Burrows, Matthew Willett

**Affiliations:** 1 School of Sport and Exercise Sciences, University of Birmingham, Birmingham, UK; 2 Sports Science and Medicine, Derby County Football Club, Derby, UK; 3 Centre or Precision Rehabilitation for Spinal Pain (CPR Spine), School of Sport, Exercise and Rehabilitation Sciences, University of Birmingham, Birmingham, UK

**Keywords:** soccer, football, exercise, groin, injury

## Abstract

**Background:**

Reduced hip adduction strength has been identified as a key predisposing factor in developing hip and groin injuries. The Copenhagen adduction programme has been shown to increase hip adduction strength in semiprofessional footballers but can cause muscle soreness. Therefore, a modified progressive Copenhagen adduction (MPCA) programme has been designed to increase hip adduction strength while limiting muscle soreness.

**Objective:**

To investigate the effect of an 8-week MPCA exercise on eccentric hip adduction and abduction strength in senior professional footballers.

**Methods:**

25 senior professional footballers completed an 8-week MPCA strengthening programme. Eccentric hip adduction (EHAD) and eccentric hip abduction (EHAB) strengths were measured. Changes in preintervention and postintervention strengths and EHAD:EHAB ratios were calculated. The statistical significance between strength changes was assessed with dependent t-tests and Wilcoxon signed-rank tests due to the distribution of the data (p<0.05). Delayed onset of muscle soreness (DOMS) and rate of perceived exertion were measured throughout the programme.

**Results:**

There were statistically significant increases in EHAD strength (24% and 25%, left and right), EHAB strength (10% and 13%, left and right) and the EHAD:EHAB ratio (12% and 10%, left and right) (p<0.01).

Professional footballers were able to complete the MPCA exercise with low levels of DOMS.

**Conclusion:**

An 8-week MPCA exercise elicited significant EHAD and EHAB strength increases with reduced levels of muscle soreness in senior professional footballers.

**What are the new findings?**

- The modified progressive Copenhagen adduction (MPCA) exercise increases eccentric hip adduction (EHAD) strength and optimises EHAD:eccentric hip abduction ratio of senior professional footballers.
- The progressive levels of the MPCA exercise prevent excessive post-training delayed onset of muscle soreness, therefore potentially enhancing compliance.
- The MPCA exercise is an easy strengthening programme that is simple and can be implemented in an applied setting.

## Introduction

The hip and groin region account for approximately 16% of injuries that occur in professional football.^1^ Adductor-related injuries contribute to 63% of these,^2^ with prolonged time missed through injury potentially costing premier-league clubs up to £750 000 per club, per season.^1^

In footballers, reduced hip adduction strength may increase the risk of developing hip and groin injuries by a factor of 4.^3^ Deficits of approximately 21% have been identified in the eccentric hip adduction strength of footballers who have hip and groin pain.^4^ Furthermore, eccentric hip adduction (EHAD) strength programmes have demonstrated reductions in hip and groin injuries.^5^ The ratios between EHAD and eccentric hip abduction (EHAB) may also impact on injury risk.^6–8^ An EHAD:EHAB ratio between 1.25 and 1.6 is thought to reduce the injury risk to footballers.^6–8^ However, Mosler *et al* did not find any association between EHAD:EHAB ratio and injury risk.^9^ Implementations of injury prevention programme in professional football is low.^10^ Post exercise muscle soreness has been linked with reduced compliance to prevention protocols.^11^ This may be a reason why prevention programmes such as the Nordic programme have been limited in professional football.^11^ Muscle soreness and fatigue require longer recovery and may decrease performance levels in footballers.^12^ Another challenge with implementing injury prevention programmes is the required dose needed to elicit the desired outcome, and when best to complete such protocols as to not impede on training and matches.

The Copenhagen adduction (CA) protocol is a graduated eccentric training regime that strengthens hip adduction and optimises the EHAD:EHAB ratio.^13^ Developed by Serner *et al*,^14^ the CA exercise is partner assisted, increasing its use in a team environment.^14^

Differing CA exercises have been shown to increase EHAD strength in footballers of 35.7%,^13^ 8.9%^15^ and 45.8%.^16^ A decrease in reported groin problems of 41% has also been seen.^5^ Muscle soreness was recorded with the median score being 0–2.^13 15 16^ There were, however, a number of athletes that had higher delayed onset of muscle soreness (DOMS) scores of 7/10^13^ and 8/10^15^ in their respective studies. The four studies mentioned earlier were completed using semiprofessional^5^ or adolescent^13 15 16^ participants. The minimal dose required and how senior professional footballers would respond to a CA exercise are still unknown. A modified progressive Copenhagen adduction (MPCA) programme starting with isometric muscle contractions and progressing to a full CA exercise has been designed to reduce the risk of DOMS and to assess if a reduced training dose negatively affects senior professional footballers.

Football staff commonly express concerns regarding injury prevention strategies, decreasing the coaching time, and training load the players can complete due to reported increased muscle soreness and fatigue.^11^

Therefore, a gap exists for an MPCA exercise that reduces comparative postexercise muscle soreness while maintaining the increases in strength, and therefore prevention of injury, in senior professional footballers.

Therefore, the aims of this study were

1. To determine the effect of an MPCA exercise on the EHAD strength and to review the EHAD:EHAB strength ratio in senior professional footballers.
2. To measure the level of post exercise muscle soreness in professional footballers who undertake the MPCA exercise.

## Methods

### Participants

Participants were recruited from a championship football club first team squad located in the East Midlands region of England by the lead investigator (GP), who was a club physiotherapist. The study was conducted from September to November 2017 at the commencement of the season, which reduced the likely impact of confounding variables, such as fatigue and cumulative injuries from match play.^1^ During the testing period, the team trained four to six times per week apart from 1 week in which they trained two times due to an international break and played 11 matches. The players did not have any experience of the CA exercise but did have experience of other adductor strengthening exercises.

Participants were included if they

- Gave their consent to participate in the study.
- Were involved in full training at the start of the trial period.

Participants were excluded if they

- Missed two or more consecutive training sessions due to injury.
- Had pain on adductor squeeze at the time of inclusion above 4/10 Numerical Rating Scale.
- Were under the age of 18.

The study was voluntary with no ramifications for players choosing not to participate. Ethical approval was obtained from the University of Birmingham Ethics Committee (reference number: CM15/06/17-1, date: 17 July 2017). This trial is reported in line with Transparent Reporting of Evaluations with Non-randomised Designs^17^ guidelines.

### Training intervention

**Figure 1:**
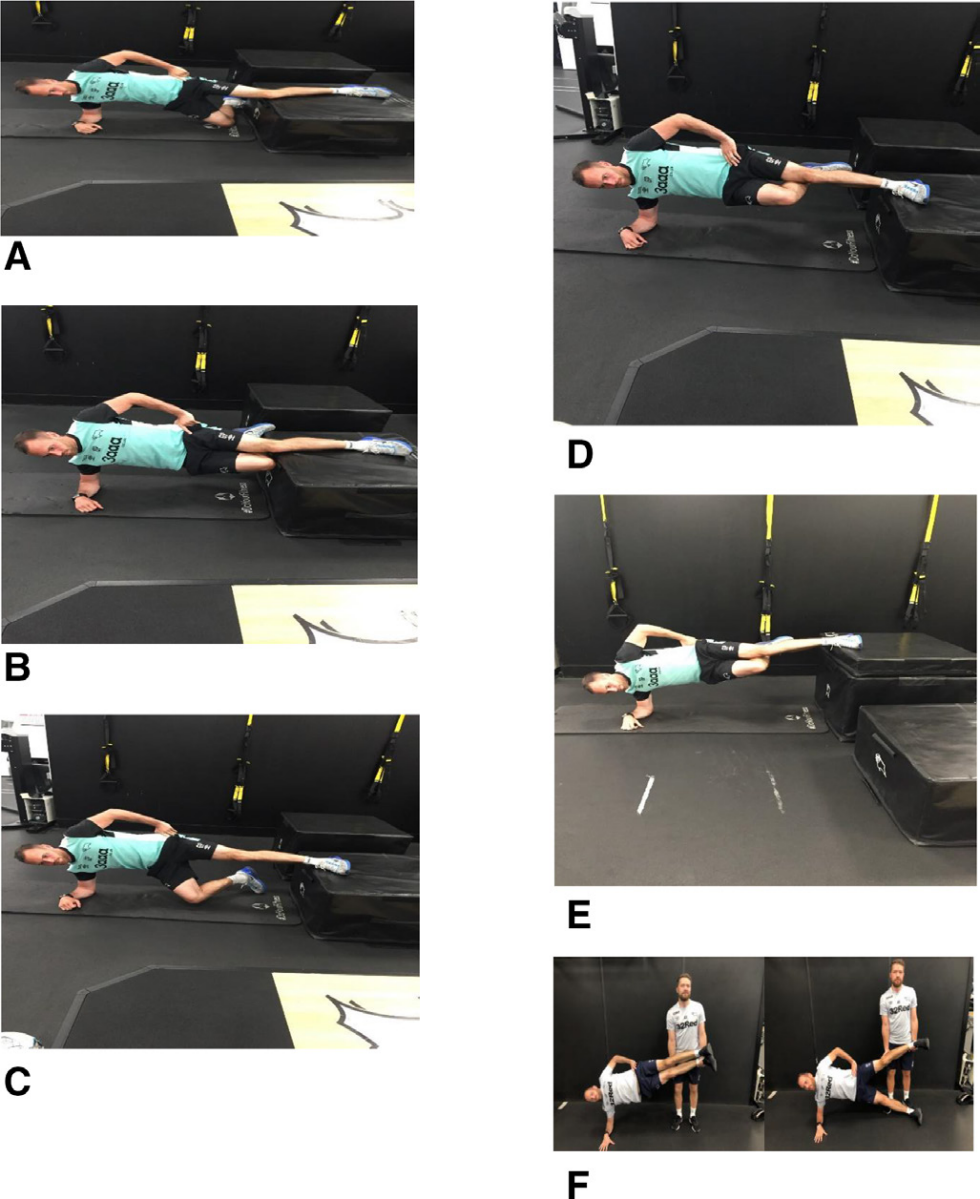
Level progressions 1–6: (A) level 1, (B) level 2, (C) level 3, (D) level 4, (E) level 5 and (F) level 6.

All MPCA sessions were conducted before football training sessions and exercises were completed on both legs. To ensure standardisation, all MPCA sessions were supervised by the same two researchers: the lead researcher (GP), a physiotherapist with experience within professional football, and a strength and conditioning coach (AB), who was part of the club’s sports science and medicine team that developed the MPCA exercise. All levels and progressions were adapted from the original CA exercise.^14^ The MPCA programme incorporated six progressive levels, specifically designed for this study, from an assisted isometric adduction hold (level 1), progressing incrementally to a full CA exercise (level 6). Level 1 is a submaximal isometric contraction; it had the lowest post exercise muscle soreness and rate of perceived exertion (RPE) when piloted with the sports science and medicine staff. Participants had to be able to perform each exercise to the satisfaction of one of the researchers before progressing to the next level (table 1). Needing a break when doing an isometric hold or being unable to control the eccentric element of the CA exercise is a reason to repeat the current level. The MPCA exercise was completed over an 8-week period. Participants completed two sessions per week, with difficulty and volume progressing incrementally. Participants progressed from two to three sets and from 6 to 10 repetitions, dependent on which level they were completing (table 1 and figure 1).

**Table 1:** Progressions in sets and repetitions through the exercise programme

Target week	Sessions	Sets per side	Repetitions	Time under tension per repetition (s)	Total time under tension (per leg, s)	Original CA exercise level
1	2	2	6	20	240	Level 1
2	2	2	6	20	240	Level 2
3	2	3	6	20	360	Level 3
4	2	3	8	20	480	Level 4
5	2	3	8	20	480	Level 5
6	2	3	6	3 s concentric 3 s eccentric	108	Level 6 (full CA)
7	2	3	8	3 s concentric 3 s eccentric	144	Level 6 (full CA)
8	2	3	10	3 s concentric 3 s eccentric	180 s	Level 6 (full CA)

Levels 1–5 are isometric muscle contractions progressing in difficulty. Level 6 is a traditional CA exercise and is an eccentric/concentric exercise shown to prevent groin problems,^5^ increase EHAD strength and EHAD:EHAB ratio.^13^

Level 1: participants performed a supported isometric adduction hold off a 30 cm box in a short-lever side-lying position. The participants then raise their pelvis from the floor, keeping their lower knee on the ground for support (figure 1A).

Level 2: participants lifted their supporting leg and brought their knees together (figure 1B).

Level 3: participants progressed to a long lever-supported isometric hold with their foot becoming the contact point for the hold. They then lifted their pelvis, keeping their lower leg foot on the floor for support (figure 1C).

Level 4: participants lifted their supporting leg and brought their knees together in a long-lever position (figure 1D).

Level 5: the box height was increased to hip height as determined by the original CA exercise^14^ (figure 1E).

Level 6: participants used a partner to perform a dynamic eccentric exercise. This involved their partner holding the upper leg at hip height and supporting the ankle and knee joints and the participant lowering their pelvis towards the ground over a period of 3 s. The participant then lowered their leg while supporting themselves and returned to the start position^14^ (figure 1F).

CA, Copenhagen adduction; EHAB, eccentric hip abduction; EHAD, eccentric hip adduction.

### Outcome measures

Descriptive data on participants included age, weight, body fat % and dominant kicking foot. EHAD and EHAB strengths were assessed using a standardised protocol^8^ with a hand-held dynamometer (HHD) (Powertrack II Commander; JTECH Medical, Utah, USA), which has demonstrated high intratester reliability (EHAD: Intraclass Correlation Coefficients 0.91 (0.70–0.98), SEM=6.3%; EHAB: ICC: 0.86 (0.53–0.96), SEM=5.1%).^8^ Testing was done in a side-lying position, and the lower leg was adducted off the ground against the HHD superior to the medial malleolus. The tester applied increasing perpendicular force onto the HHD over a 3 s period to the point where the participants’ contractions were overpowered. Eccentric abduction was tested in the same position, with the upper leg strength measured.^8^ The participants were tested three times on each leg and the mean value was calculated. The participants had 1 min between tests, and adduction was tested first to avoid a fatiguing effect.^13^ No encouragement or feedback was given until they completed all tests, keeping the procedure standardised and repeatable. The participants were assessed at baseline and 3–6 days after the 8-week training programme.^13^ Participants were tested in a rested state, having had a recovery day before testing, ensuring fatigue did not affect the results.^13^ All dynamometry testing was conducted by the lead investigator, who had four familiarisation sessions totalling eight practice hours prior to the testing period.

The participants’ post session delayed onset of muscle soreness (DOMS) in their adductors was measured using a numerical rating scale, ranging from 0 to 10, which has been used in previous studies.^13 16 18^ DOMS scores were taken 24 hours after the session had been completed.^13^ Post exercise DOMS has been shown to reduce participant compliance.^11 19^ Furthermore, progressing exercise load when severe DOMS is present may increase DOMS further and may affect players’ short-term ability to train. Therefore, a post session DOMS score below 5 was used as a key criterion for level progression within the MPCA exercise.

RPE was measured after each session, using the Borg CR10 scale,^20^ which is a numerical rating scale from 0 to 10 and has a moderate reliability in football (r=0.71, p<0.001).^20^ All participants were familiar with DOMS and RPE scales prior to the trial as they are used after each football training session.

Compliance was achieved if the participant completed all exercises in that session. Each participant needed to complete at least 70% of the sessions to be included in the study.^13^

### Statistical analysis

The data on participant strength changes were assessed for normality using a Shapiro-Wilkes test. Changes in right EHAD, left EHAD and left EHAB showed normally distributed data, but right EHAB showed non-normally distributed data. Therefore, mean differences of preintervention and postintervention in EHAD, EHAB strength and EHAD:EHAB ratio were assessed using both a non-parametric Wilcoxon signed-rank test and parametric dependent sample t-tests (baseline vs follow-up) to see if the results changed based on the statistical tests used.

All participants had a compliance over 70%; therefore, all data were included. The scores were standardised to Nm/kg to enable comparison between other studies.^5 7 13 15 21^ The minimal relevant difference (MIREDIF) for a change in adductor and abductor strength was set at 15%. A change of 15% is a realistic and clinically significant strength gain following an 8-week strength programme and is in accordance with previous similar studies.^13 21^ Statistical significance was set at p<0.05, and all analysis was conducted on the Statistical Package for Social Sciences software V.25.

## Results

### Description of sample

Twenty-five male professional footballers were initially recruited and gave their consent to participate. Six players were excluded from the study due to player transfer, and two sustained an unrelated injury during the study and so were withdrawn. Seventeen players completed the study with a compliance rate of 92.6%. The descriptive details of the participants can be seen in table 2.

**Table 2:** Descriptive data

	Average age (years) (range)	Average weight (kg) (range)	Average body fat (%) (range)	Dominant kicking foot
Participants	27.4 (20–35)	84.4 (75.0–95.5)	8.4 (5.8–12.7)	11 right, 6 left (65% right, 35% left)

### DOMS and RPE data

**Figure 2:**
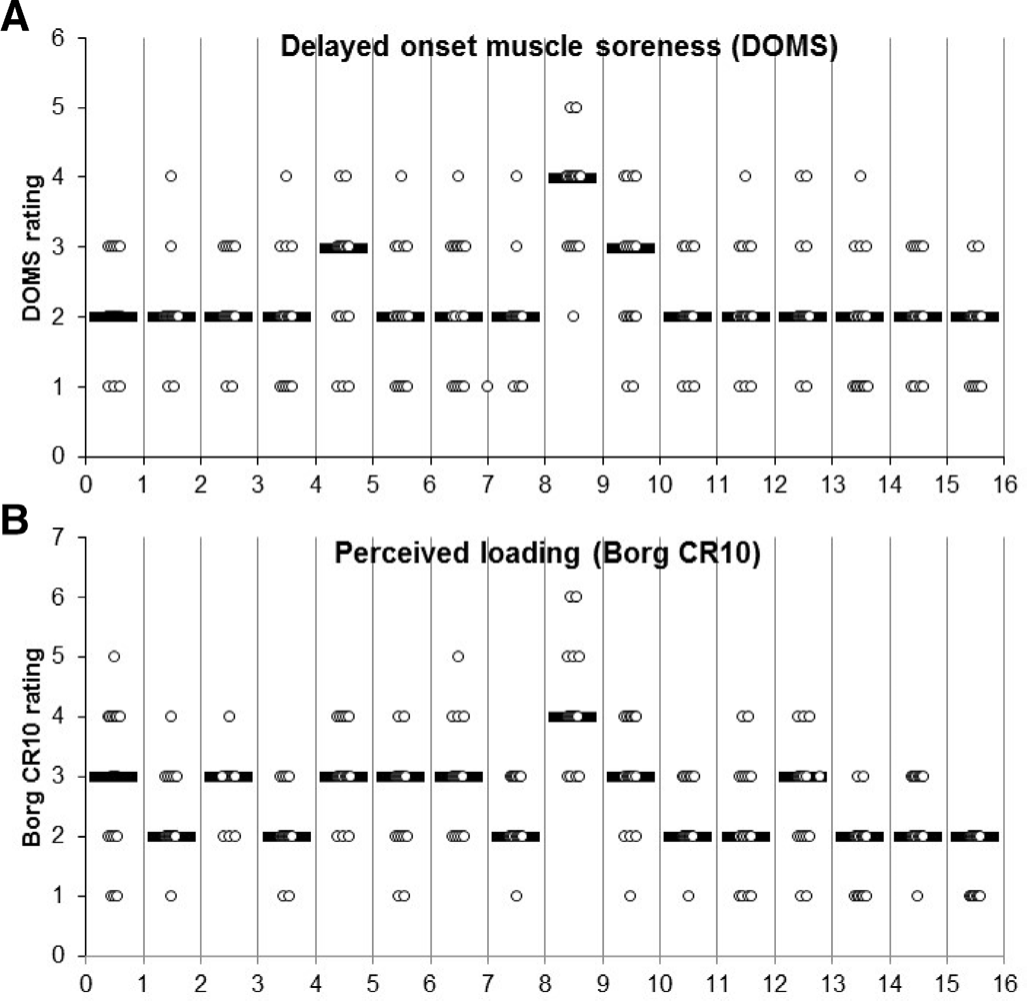
(A) DOMS following each session on a numeric rating scale (0–10). Circles indicate individual ratings. Black bars indicate mean scores.^22^ (B) Perceived loading following each session measured with Borg CR10. Circles indicate individual ratings. Black bars indicate mean scores.^22^ DOMS, delayed onset of muscle soreness.

Due to DOMS scores above 5 and suboptimal technique, two players repeated level 5. After repeating level 5, both participants progressed to level six the following week. The participants rated level 6 easier than level 5, which may be due to a decrease in time under tension. DOMS scores had a mean of 2.2 and ranged from 1 to 5 (figure 2A).^22^ RPE scores had a mean of 2.6 and ranged from 1 to 6 (figure 2B).^22^ No adverse effects of the training programme were reported.

### Effects of training intervention

**Figure 3:**
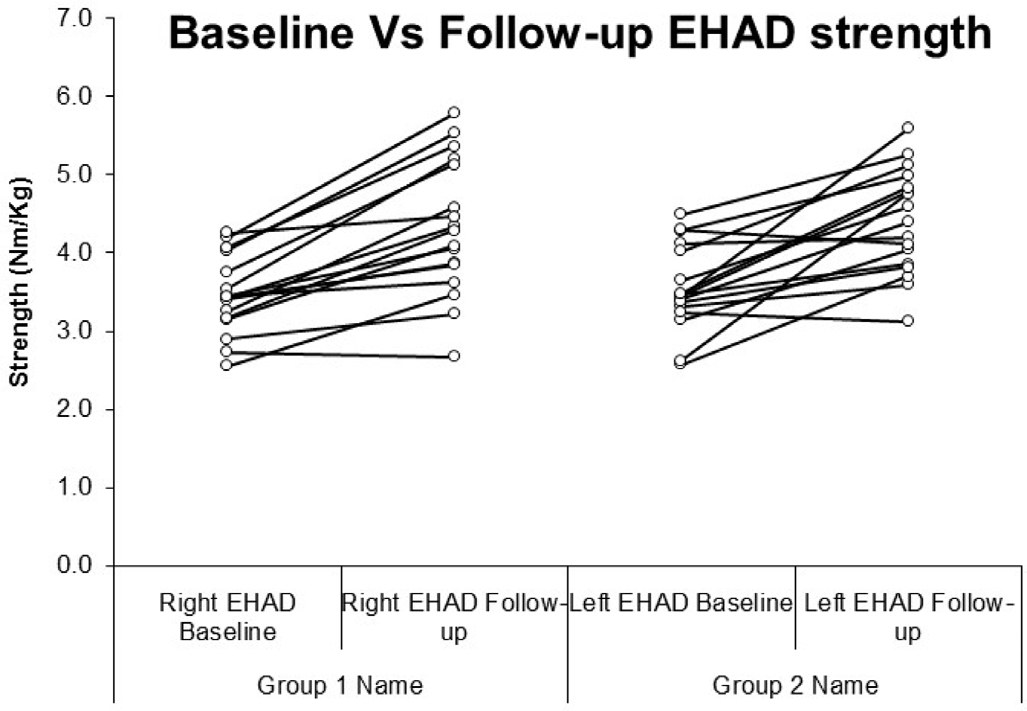
Graphs to show the improvements made in eccentrimaking the results clinically significantc strength from baseline to follow-up in Nm/kg. EHAD, eccentric hip adduction.

Both Wilcoxon signed-rank and dependent t-tests showed a significant change for all strength measurements. A statistically significant increase (p<0.01) was seen in EHAD strength bilaterally. The right-side EHAD increased by 25% (3.46 (±0.49) to 4.32 (±0.86)), and the left side increased by 24% (3.55 (±0.53) to 4.40 (±0.64)). EHAD increases were greater than 15% and were therefore above the MIREDIF, making the results clinically significant (table 3 and figure 3). A statistically significant increase (p<0.01) was seen in EHAB strength bilaterally. The right side increased by 13% (3.08 (±0.55)–3.5 (±0.67)) and the left side by 10% (3.17 (±0.43) to 3.5 (±0.64)), respectively (table 3 and figure 3). A statistically significant increase (p<0.01) was observed in the EHAD:EHAB ratio bilaterally; the right side increased by 10% (1.12 (r±0.51)–1.24 (±0.75)), the left side increased by 12% from (1.12 (±0.49)–1.26 (±0.65)). 9 (table 3).

**Table 3:** Baseline versus follow-up eccentric strength scores (in Nm/kg) (±1 SD) (95% CI) (p>0.01 for all data sets) and EHAD and EHAB strengths measured

	Baseline EHAD	Follow-up EHAD	Mean % change	Baseline EHAB	Follow-up EHAB	Mean % change	Baseline EHAD:EHAB ratio	Follow-up EHAD:EHAB ratio	Mean % change
Right leg	3.46 (±0.49) (3.21 to 3.71)	4.32 (±0.86) (3.88 to 4.76)	25% increase	3.08 (±0.55) (2.80 to 3.36)	3.5 (±0.67) (3.16 to 3.84)	13% increase	1.12 (±0.51) (0.86 to 1.38)	1.24 (±0.75) (0.85 to 1.63)	10% increase
Left leg	3.55 (±0.53) (3.28 to 3.82)	4.4 (±0.64) (4.07 to 4.73)	24% increase	3.17 (±0.43) (2.95 to 3.39)	3.5 (±0.64) (3.17 to 3.83)	10% increase	1.12 (±0.49) (0.87 to 1.37)	1.26 (±0.65) (0.93 to 1.59)	12% increase

EHAB, eccentric hip abduction; EHAD, eccentric hip adduction.

## Discussion

### Sample

Seventeen footballers in the English Championship Football League participated and completed the same training schedule. All sessions were part of the activation completed before on pitch training, which enabled high compliance. A larger sample incorporating multiple football clubs from different leagues could have strengthened the sample. Although similar results have been observed in subelite under 19 football teams,^13^ it is not known if female footballers or athletes from differing sports would gain similar benefits.

### Strength changes and the literature

The MPCA exercise demonstrated significant strength changes in EHAD bilaterally, which were above the MIREDIF of 15% commonly used in the literature. This is in line with other strengthening studies with an 8-week duration.^13 15 16 21 23^ Although the baseline EHAD strength was greater than normative data values measured for professional footballers,^7^ the MPCA exercise was still able to elicit strength changes of 25% over an 8-week programme. This suggests the MPCA exercise was challenging enough for participants to use in season, without negatively affecting training sessions or competitive matches. The minimal exercise dose (ie, number of sessions, sets and repetitions of each exercise) needed to elicit similar strength changes is not known. However, the current study has shown a lower and progressive exercise dose can elicit strength changes that are comparable with other studies, while reducing post-training DOMS scores and maintaining high compliance rates. The fact that sessions were completed prior to training may be a reason why strength gains have been seen while completing a lower dose of exercise.

Previous studies have linked strength levels with reduced injury prevalence,^7^ and one study showed that athletes that completed a CA programme had reduced groin problems by up to 41%.^5^ Therefore, CA training may reduce adductor-related injury risk in footballers. However, further research is needed to corroborate this in the professional setting. A review of adductor-related injury incidence following the MPCA exercise would be of use to further support the current evidence.

Although the CA exercise is a hip adduction strengthening exercise, abduction strength gains have been seen. This is due to an activity of 48% of maximal voluntary isometric contraction through the hip abductors, which has been shown using surface electromyography activity.^14^ This may be of additional benefit due to a further indicator of injury risk being the EHAD:EHAB ratio.^6^ The mean baseline EHAD:EHAB ratio in this study was 1.12, which increased to 1.24 right and 1.26 left, respectively. An EHAD:EHAB ratio between 1.2 and 1.6^6^ is viewed as optimal to reduce injury. Therefore, the MPCA exercise may reduce the adductor-related injury risk in elite male footballers.

Without the use of a control group, definitive conclusions cannot be made. Throughout the study, the players completed their ‘normal’ strengthening programme in addition to the MPCA exercise. This included exercises such as squats, hip thrusts, Romanian deadlifts and assisted Nordic exercises, among others. Specific adductor strengthening exercises were also not controlled for, and some participants may have completed additional adductor strengthening exercises. It could be the case that the increases in strength may be attributed to the players normal programme. However, the strength gains are seen across the participants, and the MPCA exercise was the only specific adductor strengthening exercise done.

One constraint of the current protocol is the need for equipment in the form of a box or a bench. However, the equipment needed is inexpensive and common among sports teams. Another limitation of the current protocol is the concern that level 5 could cause potential stress at the medial knee. In the current study, this was not reported by any participants and did not affect the compliance rate. A further limitation of the study was the decision to use a mean of three strength tests. This is a minor change to the testing protocol that was followed, which included piloting. However, the amount of error is not clear based on previous studies^7 15^ and may influence the reproducibility of the study.

The current MPCA exercise is focused on the adductors. In a real-world setting, football clubs require a prevention programme for all common injuries in football. It is therefore proposed that the MPCA exercise form part of a prevention programme to reduce the overall risk of injury to a professional footballer.

## Conclusion

An MPCA exercise has been shown to increase EHAD strength and to optimise EHAD:EHAB strength ratio in professional footballers’ midseason. Postintervention EHAD strength increases and EHAD:EHAB strength ratios met the threshold that have been associated with reduced risk of groin problems and adductor-related injuries.^3–8^ However, this study did not have a long enough follow-up to accurately measure the injury prevention effect. Therefore, future research should focus whether the MPCA exercise can prevent adductor-related injuries in senior professional footballers and athletes in other sports.

DOMS and RPE scores were low throughout the study, suggesting the MPCA exercise can be incorporated into football training programmes without negatively affecting performance.

The small study size may limit extrapolation of the results to other populations. However, there have been other similar small studies completed on differing populations, such as semiprofessional, or development U19 footballers, which have had similar results. This may infer the protocol could be of benefit to all levels of football. However, further assessment of the MPCA is needed to review the benefit to female footballers or athletes from other sports.
